# Newer Biomarkers in Gallbladder Carcinoma: A Scoping Review

**DOI:** 10.7759/cureus.75142

**Published:** 2024-12-05

**Authors:** Amitabh Kumar Upadhyay, Deb Sanjay Nag, Sanghamitra Jena, Neetesh Sinha, Dona Lodh

**Affiliations:** 1 Medical Oncology, Tata Main Hospital, Jamshedpur, IND; 2 Anesthesiology, Tata Main Hospital, Jamshedpur, IND; 3 Surgical Oncology, Tata Main Hospital, Jamshedpur, IND

**Keywords:** biomarkers, carcinoma, epigenomics, gallbladder neoplasms, genetic markers, genomics, local, neoplasm recurrence, prognosis, tumor

## Abstract

Biomarkers have the potential to play a crucial role in managing gallbladder cancer post-surgery. They can identify patients more likely to experience a recurrence, allowing oncologists to tailor a more intensive surveillance plan and consider additional therapies. Some biomarkers can even predict how well a patient will respond to specific chemotherapy or targeted treatments. By monitoring these biomarkers, clinicians can track how effective the ongoing treatment is and detect any signs of early recurrence. Various biomarkers, like tumor markers, genetic markers, and genomic and epigenetic markers, are being investigated. The goal is to find the most reliable and accurate biomarkers to enhance patient care and outcomes. Integrating biomarker data into treatment plans can help personalize therapy and make better informed decisions. By identifying which patients are likely to benefit from specific treatments, biomarkers have the potential to improve long-term survival rates significantly. This scoping review discusses newer biomarkers in gallbladder carcinoma; some of them are in clinical use, while most of them are used in research settings. This provides a broad insight to practicing clinicians about the present biomarkers and the futuristic biomarkers.

## Introduction and background

Gallbladder cancer (GBC), although relatively rare, is a highly aggressive disease with poor survival rates, making it a significant concern in oncology. Affecting 2.3 per 100,000 individuals globally, GBC is often diagnosed at advanced stages due to its asymptomatic onset and rapid progression [1,2]. Worldwide, GBC accounts for 1.2% of all cancer diagnoses and is the 22nd most common cancer, but 1.7% of all cancer deaths make it the 17th most deadly malignancy [1,2]. Nevertheless, it accounts for over 80% of biliary tract malignancies and ranks sixth among malignancies affecting the digestive system [1-3]. Risk factors comprise obesity, family history, viral hepatitis, and, notably, the presence of gallstones and chronic cholecystitis [4]. Asymptomatic onset and rapid disease progression characterize GBC, often leading to diagnosis at advanced stages [4]. To date, surgical resection is the only definitive curative treatment. However, with a 60% recurrence within five years, long-term outcomes remain unsatisfactory even following surgical resection [4,5]. This late diagnosis limits treatment options, and even after surgery, recurrence rates remain alarmingly high, with only a 5% five-year survival rate [4,5].

Determining the prognostic indicators to predict outcomes for non-metastatic GBC patients after surgery is crucial for developing possible adjuvant therapy and follow-up strategies. Additionally, advanced tools like nomograms, which integrate clinical and molecular data, improve our ability to predict patient outcomes and tailor treatment plans. Prognostic nomograms have demonstrated remarkable efficacy in various types of cancer, surpassing the predictive capacity of traditional staging systems [5,6]. It is possible to predict and see how many people will survive using a nomogram that looks at lymph node metastasis, degree of tissue differentiation, perineural invasion, and extrahepatic bile duct invasion. It can help doctors make treatment plans that are more effective for each patient [7].

To address these challenges, researchers are turning to biomarker molecules that provide critical insights into a disease’s presence, progression, and treatment response. These biomarkers can potentially revolutionize GBC care by enabling early diagnosis, predicting outcomes, and guiding personalized therapies [8,9]. Genetic testing, on the other hand, is not the same as cancer biomarker testing because it looks for inherited genetic differences linked to cancer susceptibility, hereditary cancer, or cancer-related syndromes [10]. New research is coming out on the role of biomarkers in GBC susceptibility, diagnosis, prognosis, and treatment selection. New research is also coming out on the role of biomarkers in the prognosis of gallbladder carcinoma after radical resection, which could help predict relapse, tailor and guide therapy, and improve outcomes. This report delves into the role of biomarkers in GBC, focusing on their diagnostic, prognostic, and therapeutic potential. It also explores emerging multi-omics approaches that hold promise for enhancing our understanding of this challenging disease.

Methodology

We searched PubMed, Scopus, and Web of Science databases from January 2000 to December 2023 using the following keywords: “gallbladder cancer”, “novel biomarkers”, “genetic susceptibility”, “prognostic markers”, and “proteomics”. Boolean operators AND/OR were used to combine terms. Searches were limited to English-language articles. The manuscript included the most relevant papers related to individual subheadings. The content was summarized and written in our own language in the form of a scoping review.

## Review

Biomarkers of genetic susceptibility

To find people likely to get GBC, researchers look at things like inflammation, drug metabolism, hormones, DNA repair, apoptosis pathways, and genome-wide association studies to find specific genetic variants that make individuals more likely to get GBC in different groups. Researchers have linked certain variations in the prostaglandin-endoperoxide synthase gene to an increased risk of bile duct carcinoma. The PTGS2−1195GA genotype is one of these variants that is linked to a higher risk of GBC [11,12]. Similarly, studies have linked genetic variations in Toll-like receptors to an increased susceptibility to GBC [13]. Genetically, GBC is associated with different types of drug metabolism enzymes such as CYP1A1 and glutathione-S-transferase class mu 1 [14,15].

Germline mutations

Approximately 15% of reported cases exhibit pathogenic germline variants (PGVs) that confer a predisposition to cancer [16-19]. We identify these variants in high, moderate, and low penetrance genes. The most common pathogenic variants in high penetrance genes include BRCA1, BRCA2, CDKN2A, MLH1, MSH6, and TP53. ATM, CHEK2, HOXB13, MITF, NBN, RAD51C, and RAD51D exhibit common pathogenic variants in genes with moderate penetrance. Frequent pathogenic variants in BARD1, MUTYH, and RAD50 are frequently found in low-penetrance genes [16-19]. In a larger sense, germline genetic markers can be considered cancer biomarkers that offer information about treatment options and cancer susceptibility. Participants in clinical trials for precision therapy may be eligible if they carry PGVs [20].

Genomic/somatic alterations

Among the genes that alter most in GBC are TP53, SMAD4, NOTCH1, ERBB2 (HER2), PIK3CA, MET, PTEN, ATM, NTRK1, CDKN2A/B, STK11, EGFR, KRAS, BRAF, CTNNB1, NRAS, and CCNE1 [21-26]. TP53 is the tumor suppressor gene (TSG) that is altered in about 90% of cases. SMAD4, a protein involved in the TGF-β signaling pathway, is the next most prevalent TSG. Other TSGs that are frequently altered, accounting for 45.45% of cases, are NOTCH1 (involved in intercellular signaling) and ERBB2 (encoding a transmembrane glycoprotein in the epidermal growth factor receptor family) [21-26]. We also detect mutations in MET, which codes for proteins involved in cell growth and survival; PTEN, which is involved in the tumor immune microenvironment; and PIK3CA, which is involved in the tumor immune microenvironment, at 33.33% and 30.30%, respectively. The ATM gene, often mutated in GBC, is essential for DNA repair via the homologous recombination pathway [21-26].

Because TP53 mutations and other driver genes are mutually exclusive, their potential as prognostic markers is highlighted. Additionally, GBC dramatically changes the PI3K signaling pathway, making it a possible target for PI3K inhibitor therapy. FDA-approved medications for other cancer types can be used to treat PIK3CA mutations, ATM, ERBB2, and ERBB2 amplifications. This implies they might be the focus of upcoming GBC therapies [27]. In addition to being an FDA-approved biomarker, NTRK1 fusion is a genetic event that can predict the effectiveness of medications such as entrectinib and larotrectinib in GBC [27]. Overall survival is not favorable for patients with changes in STK11 and SMAD4 [21-26].

Half of GBC patients have at least one molecular change identified by mutations and gene amplifications, which may lead to targeted treatments for other cancer types [27]. Among these changes were microsatellite instability (MSI-High) and high tumor mutational burden (TMB-High) [28,29]. The FDA has approved pembrolizumab, also known as nivolumab plus ipilimumab, to treat all solid tumors with MSI-High or TMB-High, including GBC [30,31]. Although certain gene alterations are common in GBC, there are few clinical trials for these alterations. This emphasizes how urgently multicenter clinical trials are needed to determine these treatment targets.

Not all therapeutic approaches that target genomic mutations produce meaningful clinical results. For instance, the inhibitor PD-1/PD-L1 has been associated with a poor prognosis and acts on the programmed cell death protein-1/ligand-1 [32]. The direct predictive value of PD-L1 status on clinical outcomes has not been definitively demonstrated, although adding durvalumab, a PD-L1 protein blocker, to gemcitabine plus cisplatin has greatly increased overall survival [33].

Epigenetic variations

Epigenetic modifications are inherited through genes but do not entail modifications to the DNA sequence. These alterations significantly impact gene expression. Among the most significant ways epigenetics regulates genes are DNA methylation, chromatin changes, nucleosome location, and post-transcriptional gene regulation by noncoding RNA or miRNAs [34,35]. The primary epigenetic mechanism that prevents TSGs from functioning in many cancer types is aberrant methylation of 5′ gene promoter regions. More and more scientists agree that the main mechanism for deactivating TSGs is the DNA methylation of these promoter regions. Research has demonstrated that TSGs like P16, APC, MGMT, hMLH1, RARβ2, p73, DAPK1, DLC1, and TIMP3 are silenced in GBC [34-36].

Investigating miRNA expression has become a quickly developing area of study to comprehend the pathophysiology of various cancers [37]. Apoptosis, migration, invasion, radiosensitivity, chemosensitivity, and the phenotype of cancer stem cells are among the characteristics of cancer that are impacted by miRNAs, which scientists have discovered to be crucial modulators of gene expression [38-40]. Additionally, there is ongoing research into the potential of miRNAs as cancer biomarkers and therapeutic targets. Of the 439 miRNAs that were found to be differentially expressed, 19 exhibited significant upregulation, and 29 exhibited downregulation. miRNAs such as miR-1, miR130, miR-146, miR-182, and miR-21, which may be biomarkers for the early diagnosis of GBC and its potential diagnostic role, can be expressed in serum [38-40].

Proteomic biomarkers

Significant progress has been made in proteomics, particularly in mass spectrometry-based protein analysis, which involves a detailed analysis of every protein expressed in a cell or tissue. These advances have facilitated large-scale protein analysis, making proteomics an essential tool in contemporary research. Proteomics encompasses a variety of analyses, including structural, functional, protein-protein interactions, protein expression profiling, and protein modifications [41,42]. In cancer research, proteomics has emerged as a powerful instrument. Researchers have identified potential biomarkers and patterns of protein expression through proteomics-based technologies that significantly impact tumor prognosis, prediction, classification, and the identification of potential responders to specific therapies. Thanks to the critical insights that cancer proteomics has given into the mechanical aspects of tumor growth and metastasis, clinically relevant biomarkers and therapeutic targets have been identified [41-43].

Through proteomics techniques, we have made significant progress in understanding the fundamental biology of cancer. The altered signaling pathways within tumor cells have been clarified, improving our understanding of cancer’s biology. This knowledge has been crucial in identifying potential targets for cancer treatments and enhancing our capacity to block various pathways critical to the disease’s spread. In cancer diagnosis, protein biomarkers - many of which are based on cancer antigens, enzymes, hormones, and changes in the protein glycosylation profile, a characteristic of cancer - have proven crucial. Glycoproteins with varying expression levels in blood and tumor tissue can be helpful markers of cancer. The tumor markers CA 19-9, CA 125, CEA, and CA 242 were notably elevated in GBC patients [44].

Mac-2BP, interferon-induced transmembrane protein 1, survivin, and discoidin domain receptor 2 are some of the most recent diagnostic biomarkers [45-48]. Furthermore, various biomarkers are being employed to help doctors predict a patient’s future health. These include the neutrophil-to-lymphocyte ratio, the derived neutrophil-to-lymphocyte ratio, the platelet-to-lymphocyte ratio, the lymphocyte-to-monocyte ratio, stratifin, Dickkopf 3, eukaryotic translation initiation factor 5A2, and mesenchymal-epithelial transition [49-54]. Similarly, elevated annexin A4, decreased heat shock protein 90-beta, and dynein cytoplasmic 1 heavy chain 1 are other proteomic biomarkers associated with GBC [55]. Similarly, aminopeptidase N, C-reactive protein, filamin-A isoform X5, integrin alpha-iib, 5′-Nucleotidase isoform 2, integrin alpha-6 isoform X6, neprilysin, pyruvate kinase PKM isoform d, serum amyloid A-1 protein, serum amyloid A-4 protein, cofilin-1, alkaline phosphatase tissue-nonspecific isozyme isoform 2, Ras-related protein Rap-1b isoform 4, dipeptidyl peptidase 4 isoform X1, protein TFG isoform X2, profilin-1, and actin cytoplasmic 2 are some other newer proteomic biomarkers that are altered in various stages of GBC [56].

The following table lists the various biomarkers and classes (Table 1).

**Table 1:** Biomarkers in gallbladder cancer and implications

Category	Biomarkers	Implications
Genetic susceptibility	PTGS2−1195GA genotype, Toll-like receptors, Cytochrome P450 1A1, and Glutathione-S-transferase class mu 1	Genetic susceptibility for developing GBC
Germline mutations	High penetrance genes: BRCA1, BRCA2, CDKN2A, MLH1, MSH6, and TP53	Approximately 15% of reported cases exhibit pathogenic germline mutations leading to gallbladder cancer. These markers have potential therapeutic implications.
	Moderate penetrance genes: ATM, CHEK2, HOXB13, MITF, NBN, RAD51C, and RAD51D	
	Low penetrance genes: BARD1, MUTYH, and RAD50	
Somatic/genomic mutations	TP53, SMAD4, NOTCH1, ERBB2 (HER2), PIK3CA, MET, PTEN, ATM, NTRK1, CDKN2A/B, STK11, EGFR, KRAS, BRAF, CTNNB1, NRAS, CCNE1, tumor mutational burden, and MSI	Acquired somatic mutations detected on tumor tissue have diverse potential therapeutic implications.
Epigenetic variations	miRNAs (miR-1, miR130, miR-146, miR-182, miR-21, etc.)	The miRNAs have been recognized as vital regulators of gene expression, modulating various cancer-related characteristics.
Proteomic biomarkers	Tumor markers CA 19-9, CA 125, CEA, and CA 242. Newer markers like Mac-2-binding protein (Mac-2BP), Survivin, Discoidin domain receptor 2, Interferon-induced transmembrane protein 1, Stratifin, Eukaryotic translation initiation factor 5A2, Dickkopf 3, neutrophil to lymphocyte ratio, derived neutrophil to lymphocyte ratio, platelet to lymphocyte ratio, lymphocyte to monocyte ratio, mesenchymal-epithelial transition, Annexin A4, Heat shock protein 90-beta, Dynein cytoplasmic 1 heavy chain 1, Aminopeptidase N, C-reactive protein, Filamin-A isoform X5, Integrin alpha-iib, 5′-Nucleotidase isoform 2, integrin alpha-6 isoform X6, Neprilysin, Pyruvate kinase PKM isoform d, Serum amyloid A-1 protein, Serum amyloid A-4 protein, Cofilin-1, Alkaline phosphatase tissue-nonspecific isozyme isoform 2, Ras-related protein Rap-1b isoform 4, Dipeptidyl peptidase 4 isoform X1, Protein TFG isoform X2, Profilin-1, and Actin cytoplasmic 2	Newer proteomic biomarkers with diagnostic and prognostic implications

Emerging technologies

Researchers worldwide have increasingly used multi-omics techniques with patient samples for translational research in recent years. This requires integrating data from several omics techniques, such as proteomics, genomics, transcriptomics, and metabolomics, to gain comprehensive insights [57-59]. In contrast to individual analyses, multi-omics analyses produce vast datasets that provide crucial insights into the complex mechanisms underlying disease pathophysiology. This approach dramatically advances the diagnosis and creation of disease treatments. The continued use of multi-omics approaches is expected to have a powerful influence on translational research, particularly in cancer biology [57-59].

Practical challenges and future directions

Currently, genetic testing is advisable for selective patients. Similarly, genomic alterations are crucial in our decision-making in metastatic settings and help us decide on targeted therapies. The usage of most of the newer biomarkers, like miRNAs and proteomic biomarkers, is very limited in research settings. There is minimal consensus regarding their diagnostic, predictive, and prognostic implications. These biomarkers can potentially evolve into valuable biomarkers with defined diagnostic, predictive, and prognostic implications in the future.. 

## Conclusions

The array of GBC biomarkers is extensive, but their clinical application in treatment is an area that continues to be researched and remains an unmet necessity. Currently, genomic biomarkers are in clinical use, as they help us make therapeutic decisions and select targeted therapies. Genetic biomarkers are used in selective cases with familial association, and they help us in genetic counseling and the selection of targeted therapies in some cases. The tumor markers, CA 19-9, CA 125, and CEA, are in routine practice, while newer proteomic and epigenetic biomarkers are primarily limited to research settings. Many of the newer biomarkers are likely to come into clinical practice in the near future. Similarly, genetic susceptibility markers are mostly limited to population-level research settings. Researchers worldwide increasingly use multi-omics methodologies incorporating data from diverse omics approaches, which has enormous potential in oncology. Clinicians must remain abreast of these advancements to enhance patient outcomes. In the interim, utilizing nomograms for treatment strategizing and prognostication is highly advisable.
